# The Scottish Medical Imaging Archive: 57.3 Million Radiology Studies Linked to Their Medical Records

**DOI:** 10.1148/ryai.220266

**Published:** 2023-09-27

**Authors:** Rob Baxter, Thomas Nind, James Sutherland, Gordon McAllister, Douglas Hardy, Ally Hume, Ruairidh MacLeod, Jacqueline Caldwell, Susan Krueger, Leandro Tramma, Ross Teviotdale, Kenny Gillen, Donald Scobbie, Ian Baillie, Andrew Brooks, Bianca Prodan, William Kerr, Dominic Sloan-Murphy, Juan F. R. Herrera, Edwin J. R. van Beek, Parminder Singh Reel, Smarti Reel, Esma Mansouri-Benssassi, Roy Mudie, Douglas Steele, Alex Doney, Emanuele Trucco, Carole Morris, Robert Wallace, Andrew Morris, Mark Parsons, Emily Jefferson

**Affiliations:** From the EPCC, University of Edinburgh, Edinburgh, Scotland, United Kingdom (R.B., A.H., R.M., D.S., A.B., B.P., W.K., D.S.M., J.F.R.H., M.P.); Health Informatics Centre (T.N., J.S., G.M., D.H., S.K., L.T., R.T., K.G., P.S.R., S.R., E.M.B., R.M., E.J.), Division of Imaging Science and Technology (D.S.), Division of Population Health and Genomics (A.D.), and Department of Computing (E.T.), University of Dundee, Dundee DD1 4HN, Scotland; Public Health Scotland, Edinburgh, Scotland (J.C., I.B., C.M., R.W.); University of Edinburgh Brain Research Imaging Centre, Edinburgh Imaging, Queen's Medical Research Institute, Edinburgh, Scotland (E.J.R.v.B.); Health Data Research UK, London, England (A.M., E.J.).

**Keywords:** MRI, Imaging Sequences, Ultrasound, Mammography, CT, Angiography, Conventional Radiography

## Abstract

**Keywords:** MRI, Imaging Sequences, Ultrasound, Mammography, CT, Angiography, Conventional Radiography

Published under a CC BY 4.0 license.

See also the commentary by Whitman and Vining in this issue.

**Summary**

The Scottish Medical Imaging (SMI) Archive is a collection of population-based, medical radiology images from real patient records for use in health care research and the development or validation of artificial intelligence algorithms within the Scottish National Safe Haven. The images are linkable to other routinely collected electronic health care records (such as hospital, prescribing, birth, and death data).

**Key Points**

■ The Scottish Medical Imaging Archive is a collection of population-based, routinely collected medical radiology images available to researchers for use within the nationwide Scottish safe.
■ This archive provides access to “analytics-ready” extracts for images between January 1, 2010, and August 31, 2018, which can be used for health care research and the development or validation of artificial intelligence algorithms.
■ This archive is fully compliant with the U.K. Data Protection Act and preserves patient confidentiality using pseudonymization techniques.
■ An archive of 57.3 million radiology studies linked to their medical records from the whole Scottish population has been made available for research within a trusted research environment on a cost recovery basis.
■ The end-to-end service to access the data is provided through the electronic Data Research and Innovation Service team *(https://www.isdscotland.org/Products-and-Services/eDRIS/Scottish-Medical-Imaging-Service/)*.
■ An open-source software platform hosts and manages the data, providing the capability to extract relevant images based on complex cohort definitions and to capture research annotations and ground truth.

## Introduction

Using clinical images for research and linking them to other routinely collected clinical data is challenging because of the following reasons:

1. A clinical picture archiving and communication system (PACS) is (quite rightly) designed and optimized for clinical care rather than research. A clinical PACS makes it easy to find all images for a particular patient, but it is not designed to facilitate searching for images with particular characteristics, such as section thickness, scanning protocol, contrast agent, and patient medication.
2. Reuse of clinical images for research requires de-identification, yet identifiable data can be present in many areas of the associated Digital Imaging and Communications in Medicine (DICOM) file metadata and/or may be present within the pixel data itself and therefore “burned onto” the actual image.
3. Reuse can require approval from multiple data controllers, and the complexity of de-identification increases the risk of rejection of applications for research given the amount of work the data controller may have to do to ensure that no identifiable data are released.
4. Ground truth or reference standard data developed by one research project are not easily shared with other research projects because of both the technical and data governance challenges of doing so when data are sensitive.

We have addressed these four challenges by collecting routinely captured radiology images from the Scottish population, linking these data to longitudinal electronic health records, and building a software platform and end-to-end service to support researchers in accessing de-identifiable cohort-specific subsets of the data for research. There is a single data governance application process for accessing the resource (although the data were captured by multiple data custodians), and the software platform can store and share ground truth and annotations from one research project with another (provisional on consent to share).

## Materials and Methods

### Ethics

Public Health Scotland has a generic ethics approval for a research database. This covers the collation, linkage, and secondary use of data held by Public Health Scotland and analyzed within the secure environment of the National Safe Haven (NSH). No ethical approval was required to build this database. The Health and Social Care Public Benefit and Privacy Panel (PBPP) approved the application requesting data access to bring the data over from the national PACS into a research database to be held in the NSH.

### Data Resource Collection

Access to “analytics-ready” extracts of the archive is through the Scottish Medical Imaging (SMI) Service, part of the electronic Data Research and Innovation Service (eDRIS) team (1) within Public Health Scotland.

The SMI Archive holds some 2.47 billion DICOM images in 94.9 million series across 57.3 million studies and essentially mirrors the national PACS data archive for the entire country. It covers 36 imaging modalities, including CT, MRI, PET, structured reports, radiography, and US. These are images collected through routine clinical care across all the 14 National Health Service (NHS) health boards within Scotland, hence providing wide coverage of the Scottish population. The CT, MRI, PET, and structured report modalities (approximately 75% of the studies within the archive) have been curated and are available for researchers to access (Table 1). The remaining modalities are being curated. Detailed descriptions of the SMI fields are available from the SMI Research Dataset (2).

**Table 1:**
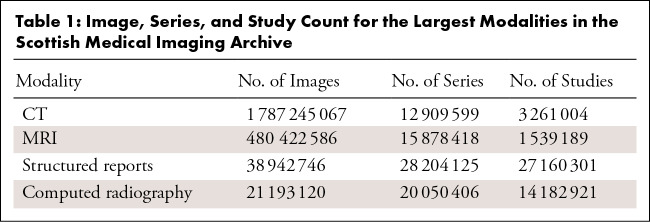
Image, Series, and Study Count for the Largest Modalities in the Scottish Medical Imaging Archive

**Figure 1:**
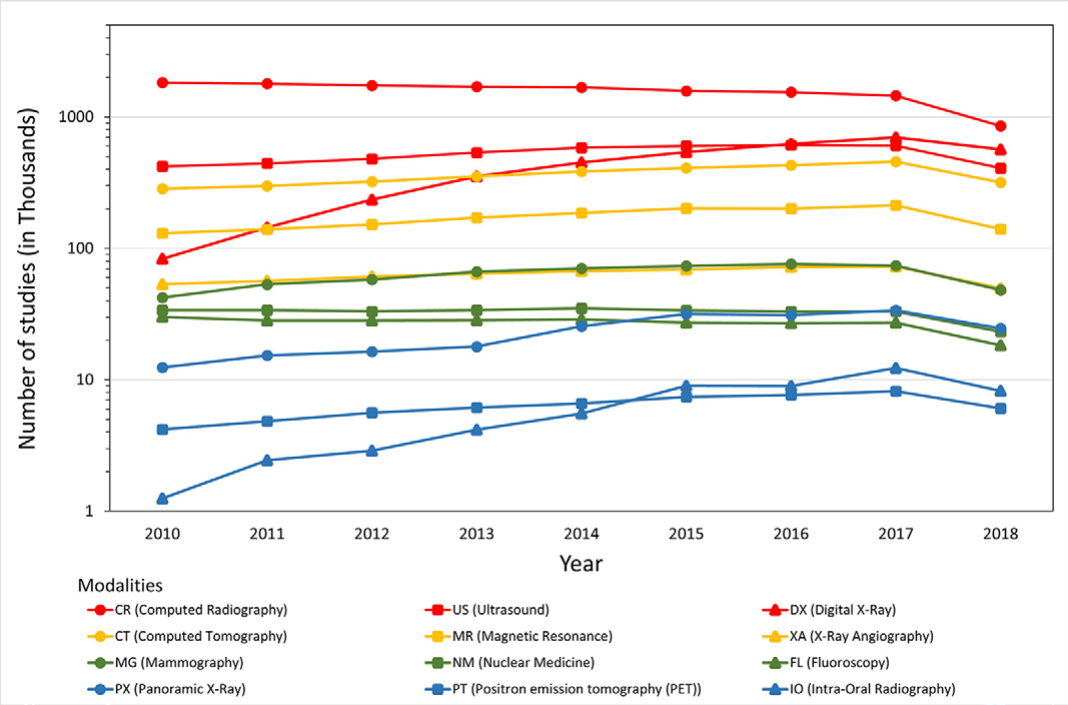
Graph shows the number of studies per modality within the Scottish Medical Imaging Archive by year of imaging. Because the data are available for only a fraction of 2018, the volume of studies for this year is less than for the previous year.

The initial dataset spans from January 1, 2010, to August 31, 2018 (process to update the archive is ongoing). Figure 1 shows the total number of DICOM studies by modality and year. Because the data are available for only a fraction of 2018, the volume of studies for the year is less than the previous year.

There are 2 182 123 female patients (13 167 604 unique study identifiers) and 2 081 040 male patients (11 265 313 unique study identifiers) recorded in the SMI Archive. Because the population of Scotland is approximately 5.5 million, this means that about 1.2 million people have never undergone a radiologic examination (approximately 22% of the population). Patient years of birth range from 1900 to 2020, with a median year of birth of 1960–1970 (aggregated data). Information on patients’ ethnicities is shown in Table 2.

**Table 2:**
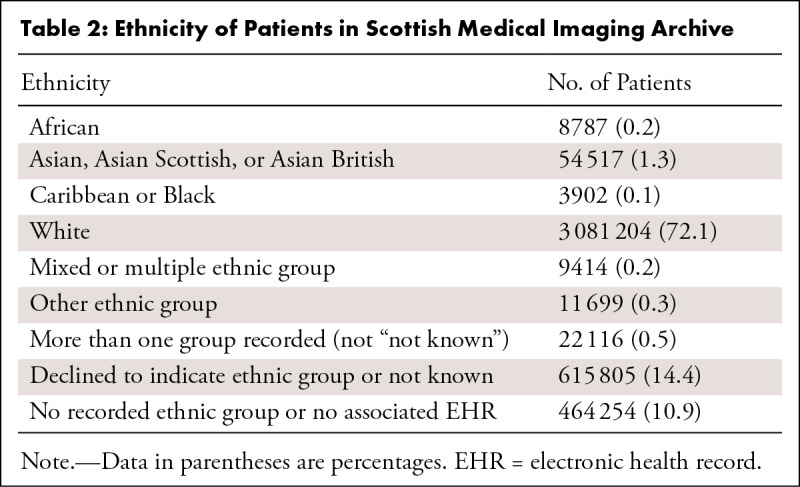
Ethnicity of Patients in Scottish Medical Imaging Archive

Of patients with scans, 94.2% have associated longitudinal health care records. As might be expected, the percentage of patients who appear in different longitudinal health care records differs depending on the type of record (eg, 16.8% of patients have linked cancer records, 22.3% of patients have linked maternity-related records, and 92.4% of patients have linked hospital outpatient records).

## Resulting Dataset

### Data Resource Use: Study Dataset Extraction and Cohort Assembly

We have worked for the past 7 years to build a suite of software tools for hosting and managing more than 3 petabytes of data from different sources (both imaging data and longitudinal electronic health records) (3). An end-to-end service to extract researcher-specified, relevant cohorts of data from the large data resources and share these with researchers within a secure environment is provided by eDRIS. Data extracts can be requested for a given study based on DICOM tag data, routinely collected clinical data (eg, prescribing, hospital admissions), the researcher's existing cohort, and/or the results of natural language processing classification of structured reports. Extraction is performed by an eDRIS analyst using tools specially developed by PICTURES (4) to derive the study population from clinical data (5) (eg, age >40 years with any lung cancer diagnosis). The study cohort can be further refined by the analyst (eg, patients with CT chest images showing lung nodules) and can include concepts derived from the processing of any structured reports associated with the images in the study. We are developing support for cohort assembly from pixel data directly (eg, by running an image classification algorithm).

**Figure 2:**
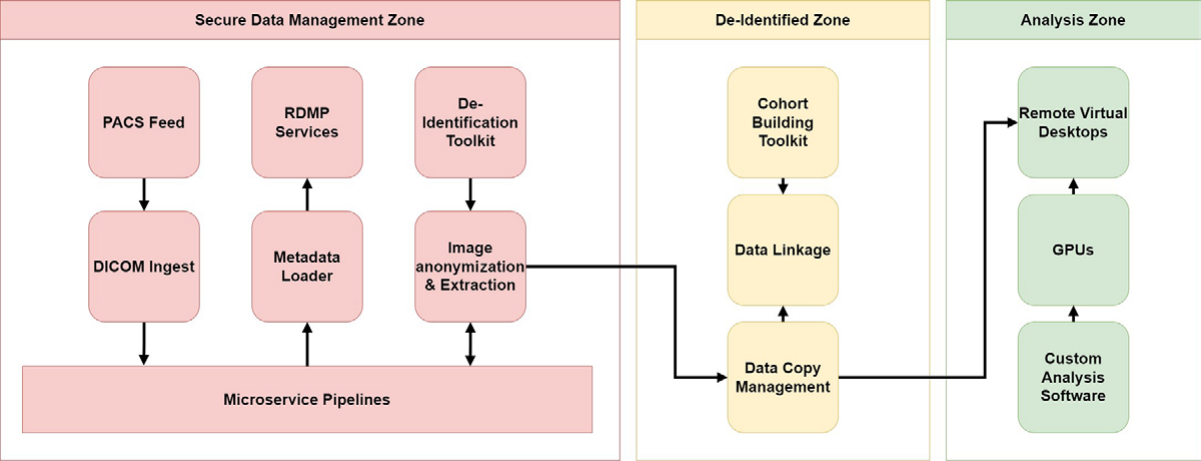
High-level architecture of Scottish Medical Imaging platform. There are three zones: the Secure Data Management Zone, the De-identified Zone, and the Analysis Zone. Within the Secure Data Management Zone, an identifiable copy of all the raw DICOM files is hosted along with a database containing a copy of the DICOM tag metadata. A microservices pipeline manages all processing, including transferring metadata to the De-identified Zone and images to the Analysis Zone. Within the De-identified Zone, the electronic Data Research and Innovation Service (eDRIS) team builds the cohorts required for each research project and links data. The Analysis Zone is where researchers log onto a virtual environment to carry out their research on a copy of the data provided for their specific project. The unidirectional arrows show that the data travels in only one direction. However, metadata and processes or tools can travel back to the other zones to support future cohort-building capabilities. DICOM = Digital Imaging and Communications in Medicine, GPU = graphics processing unit, PACS = picture archiving and communication system, RDMP = Research Data Management Platform.

Nind et al (3) provided an architectural description of the open-source software tools (5,6) supporting data management and cohort extraction. In summary, there are three zones within the architecture, as shown in Figure 2.

### Data Linkage

SMI image extracts can be linked at an individual level to other health care datasets available within the NSH using the Scottish community health index number, a unique numeric identifier allocated to each patient on first registration with the Scottish health care system (5). Such health care datasets include hospital records (Scottish Morbidity Records [SMR]00 and SMR01), maternal and neonatal records (SMR02), the Scottish cancer registry (SMR06), prescribed and dispensed drugs (6), and mortality records; a full list is available from the Scottish National Data Catalogue (7). Other externally produced datasets from clinical trials, local health boards, disease registries, and nonhealth administrative data can also be linked.

### Data Resource Access

Researchers can request pseudonymized, linkable SMI data by contacting the eDRIS team. Each research proposal is assigned an experienced research coordinator, who will guide researchers through the selection of appropriate data items and any limitations applicable to the proposed study, as well as guidance on the completion of the PBPP application process for approval to access NHS Scotland data.

For an application requesting images linked to other data to the PBPP, the mean time from submission to approval is 66 days. When the time taken for applicants to respond to queries from the panel is deducted, the mean approval time is 21 days. Submission to PBPP occurs when the eDRIS research coordinator, based on their knowledge and experience, agrees with the applicant that their application is of a suitable standard.

Once the PBPP approves the research study, data are extracted and provided to researchers through individual project accounts within the NSH, Scotland's national trusted research environment. Researchers can build their own software stack and import this into the NSH to be linked to the data. The NSH is a locked-down environment adhering to the five safe principles of trusted research environments: safe data, projects, people, settings, and outputs (8,9). Access to the internet is controlled, and disclosure controls are applied to any data to be exported.

To preserve patient confidentiality and ensure that all research with SMI linked data are fully compliant with the U.K. Data Protection Act (9), the eDRIS team applies pseudonymization techniques on data extracts for researchers’ use. All personally identifiable information is removed from linked datasets, and patient identifiers are replaced with unique pseudoidentifiers. SMI data are available to researchers affiliated with U.K. public sector organizations, and requests can be made to provide access to those outside the United Kingdom. For more information regarding application and costs, contact eDRIS (1). Details of the metadata can be found at the SMI Research Dataset (2).

The dataset is “open” because, given a successful PBPP application, access to the data will be provided. The PBPP application process is an independent process that reviews whether the project is in the public interest and ensures that patient confidentiality is maintained. There is no requirement to collaborate with the specific research group who developed the platform or to pay for access via a commercial company, as might be expected for a “closed” dataset. If a group wishes to develop a commercial artificial intelligence (AI) product, providing the product at a discounted price to NHS Scotland may be required because the product will have been trained on data from this population.

The dataset has been created through routine health care provision without the consent of the participants for their health data to be shared for research purposes. Because of linkage to longitudinal health care records, in many cases it is not possible to ensure that the images and associated data are fully anonymous. In such cases, it is not possible within the U.K. laws of common duty of confidentiality and the U.K. Data Protection Act (9) to make these data available for direct download; therefore, access is provided within the NSH environment. The NSH environment allows analysis and AI development, but the data itself cannot be exported. Only a specific subset of the dataset is provided to answer the specific research question (eg, foot radiographs are not provided for a study investigating or training on lung MRI examinations). The environment and data are provided on a cost recovery basis (ie, the NHS data controllers do not charge for access to the dataset, but there are associated costs to provide the services needed for the research project).

### Example Studies

The resource is live and available for access, and it is being used by many research groups. Current approved studies include the development and validation of the CT clock tool for estimating the time of ischemic stroke onset (10), whole-population automated reading of brain imaging reports in linked electronic health records (11), making retinal images from chain retail optometrists’ research ready and linkable to other data with the Scottish Collaborative Optometry–Ophthalmology Network E-research (SCONe) (12), and linking brain imaging to the cohort of professional rugby and football players (Football's InfluencE on Lifelong Health and Dementia risk: Late Outcomes and NeuroradioloGy) (13). Chest radiographs, MR images, and CT scans of hospitalized COVID-positive and COVID-negative patients were provisioned to the U.K. National COVID-19 Chest Image Database (14).

## Discussion

The SMI data resource is a population-scale, heterogeneous dataset of radiology images linked to longitudinal health care records. An end-to-end service helps researchers access subsets of the data relevant to their particular study within a trusted research environment.

There are many strengths of this resource:

1. The Scottish population is a relatively stable population with comparatively little immigration or emigration (the non-UK born population is 9.7%) (15).
2. The population is relatively unhealthy compared to other Western European countries, for example, there are no Western European countries whose females have a lower life expectancy (16).
3. The NHS provides excellent longitudinal health care records (linked by the community health index for decades). Images have been collected from different health boards using different scanner types and processes, thus providing highly heterogeneous real-world data, well suited for training robust, generalizable AI algorithms.
4. As the images are population-wide, the bias in the data is minimal, as the number of scans is proportional to the scans requested to provision routine health care.
5. The ability to link the clinical imaging data relatively easily to other datasets is highly advantageous.

There are also several limitations of this data resource:

1. The Scottish population is not ethnically diverse (approximately 96% White) (16).
2. AI models might accidentally encode personal data, which may later be susceptible to external hacking (eg, reverse engineering training data). We are researching effective disclosure control methods for AI models (17) to support extraction from the NSH, but this process is not yet implemented in production.
3. At present, no automated methods prevent three-dimensional reconstruction of a face from images of the head (governance rules prohibit this), nor are there restrictions on the extraction of images of people who have unique conditions (or uniquely identifying features like tattoos) that could allow reidentification. Cases of this nature still require substantial manual inspection by eDRIS analysts before such data can be released.
4. Stratifying images by sequence type (eg, T1-weighted images, T2-weighted images) and/or body part is a challenge with relying solely on clinically gathered DICOM tag data. We are developing methods to improve this (eg, algorithmic cross-referencing with pixel and/or structured report data).

We are working to enhance the resource with future developments including the following:

1. Additional years of data: At the time of publication, the SMI Archive is a single snapshot of all radiology images taken in Scotland between January 1, 2010, and August 31, 2018. However, we are bringing the archive up to date and setting up a regular feed to update the resource from the Scottish national clinical PACS.
2. Additional modalities of data: As researcher demand dictates and resource allows, we will process and make available additional DICOM modalities. The first addition will cover the radiography modalities (computed radiography, digital radiography, and panoramic radiography).
3. Additional DICOM tags: As researcher demand dictates and resources allow, we intend to make additional DICOM tags available as variables that can be selected as part of research cohort construction. The review process for tag promotion will consider aspects such as overall demand, future use and benefits for PHS, and future research requests.
4. Annotation and enrichment of data: Research and development work within the PICTURES program is exploring capturing annotations and ground truth from researchers. We will use this feedback to augment and enrich the archive, either as additional information for cohort creation or as additional derived data variables that can be offered to future researchers, so the annotations available for the resource increase over time. In addition, several ongoing projects are focused on classifying image subsets (eg, body part, scan type, compression detection).

In conclusion, the SMI data resource is available for international researchers and innovators to perform health care research and for the development or validation of AI algorithms. We welcome the opportunity to collaborate to enhance the capability of the resource and will respond to new user requests.
